# Assessing Depression among Older Persons with Arthritis: A Nationwide Health Status Survey

**DOI:** 10.1155/2013/968343

**Published:** 2013-07-15

**Authors:** Rajesh Nayak, Jigar Rajpura

**Affiliations:** ^1^Department of Pharmacy Administration, College of Pharmacy and Health Sciences, St. John's University, 8000 Utopia Parkway, Jamaica, NY 11439, USA; ^2^Department of Pharmacy Practice, College of Pharmacy, Purdue University, 575 Stadium Mall Drive, West Lafayette, IN 47907, USA

## Abstract

*Objectives*. This study aimed to assess the health status of a nationwide sample of elderly persons having arthritis and determine the prevalence of depressive symptomatology in this population. *Methods*. WebTV technology was utilized to administer health status and depression surveys to a nationally representative sample of 550 randomly selected older persons. Predetermined cutoff scores on Short Form-36 (SF-36) scale and Center for Epidemiological Scale for Depression (CES-D) were used to identify individuals with depressive mood. *Results*. Sixteen percent (*n* = 76) of the respondents were found to be at risk for depression. Key associations among health domains of SF-36 and CES-D variables were statistically significant and were in the expected direction. *Discussion*. The risk of depression among older adults who have arthritis is moderate. A significant decline in multiple domains of health of older persons is likely when depression coexists with arthritis. Early screening for depressive symptomatology and prompt treatment should be an essential part of arthritis management in primary care practice.

## 1. Introduction

In 2001, an estimated 70 million Americans had some form of arthritis or other rheumatic conditions, a major increase over the previous estimate of 43 million cases reported in 1997 [1]. In 1992, arthritis cost the U.S. economy about $64.8 billion dollars (about 24% was due to direct medical costs, 76% due to indirect costs from lost wages) [2]. The cost of treating arthritis and other musculoskeletal conditions in 1992 was $149.4 billion, which was about 2.5% of the Gross National Product [2, 3]. Because arthritis conditions do not usually cause death, quality of life (QOL) is a better indicator of the disease impact than mortality rates in a nonterminal disease like arthritis. In addition to the quality of life, arthritis can also negatively impact the well-being and satisfaction with life in people with severe forms of the disease [4, 5]. Debilitating nature of the disease, mainly due to chronic physical pain, can affect all aspects of a patient's life, including the physical, behavioral, social, and psychological. Consequently, an overall evaluation of health status in arthritis is viewed to be important for broadening the focus of care and improving patient outcomes beyond survival.

The adverse impact of arthritis on mental health of a patient has been documented frequently in the literature, particularly in the case of rheumatoid arthritis (RA) [6–8]. Rates of depression are noticeably higher among patients with chronic pain such as rheumatoid arthritis [9]. For persons of 65 years of age or older, depression is often correlated with a decline in physical health [10]. According to an expert estimate, about 90 percent of patients with arthritis and other rheumatic diseases experience depression [11]. However, the link between different types of arthritis and depression is unclear. Depression is often assumed to be a normal response to aging, physical pain, or other life events and is frequently overlooked as a clinical diagnosis in older people. Because depression can make an independent and additive contribution to disability accompanying arthritis, early recognition of depressive symptoms is essential for an effective management of the disease. Maintenance of good health status that is free from the burden of mental illness is crucial for producing positive health outcomes in patients affected by arthritis. Accordingly, the primary objective of this study was to comprehensively measure the health status of older adults with arthritis and to determine the prevalence of depressive symptomatology in this population.

Researchers in health services area have used Short-Form 36 (SF-36) [12], a health status survey, and Center for Epidemiological Scale for Depression (CES-D) [13], a screening tool for depression, extensively for many years. Health related quality of life (HRQOL), an outcome that is measured by SF-36, represents multiple dimensions of health that cover physical and social activities, well-being, and overall evaluation of health. The SF-36 is suitable for self-administration, computerized administration, or administration by a trained interviewer in person or by telephone to persons aged 14 and older. The reliability of the scale has been previously established to meet or exceed the minimum standards recommended for measures used in group comparison [14]. Even though SF-36 has been used routinely in the assessment of health in patient populations, with the exception of a few studies [15, 16], its utility as an instrument that screens for depression remains largely unexplored. One of the eight domains of SF-36, the 5-item mental health (MH) subscale, has been shown to be useful as a first stage screen for depression. Using the standard (0–100) scoring, a cut-point of 52 on the MH scale is often cited as best in several studies using receiver operating characteristic (ROC) analysis to screen for major depressive disorders [11, 17, 18]. Yet, to a large part, the use of SF-36 in the past has been mostly confined to the assessment of health status. 

The 20-item CES-D, the instrument originally developed for National Institute of Mental Health Studies [19], is judged among the best screening instruments for symptoms of depression in older adults [20, 21]. Its reliability and validity to detect both clinical and nonclinical symptoms of depressed mood are established for a wide range of study populations [12, 22], including older adults [13, 23, 24]. Concurrent use of SF-36 and CES-D has been reported in the literature before, but seldom with a goal to examine the utility of SF-36 as a depression screener, when a more established CES-D is also used. It can be argued that the usefulness of SF-36 would substantially increase if it could also serve as a screening tool for depression, obviating the need for a separate depression instrument. Therefore, a secondary purpose of this research was to determine whether SF-36 could be a good tool for the measurement of depression and to assess its performance relative to that of an established instrument like CES-D.

## 2. Methods

### 2.1. Sample Selection

A cross-sectional, web-based, survey research methodology with random sampling techniques was employed. Study participants were drawn from a nationwide sample of forty thousand U.S. households across 44 states. To be eligible, the respondents had to be 65 years of age or older, have arthritis, and have no clinical diagnosis of depression. SF-36 and CES-D surveys were coadministered through WebTV technology. In August 2001, proprietary Random Digit Dialing (RDD) survey procedures, developed by a New York-based commercial market research firm, were employed for screening, subject recruitment, and survey administration. The research firm employs RDD sampling for selecting telephone numbers of eligible households and invites them to join a panel. The participants are then sent a WebTV set-top box and given free internet access in exchange for completing surveys approximately once a week. A sample of about 40,000 10-digit randomly selected numbers constituted our sampling frame and was representative of the entire USA. Following the initial screening achieved through internet and/or telephone contacts to establish the participant's age and medical condition, a sample of 550 randomly selected elderly arthritis-affected individuals agreed to participate in the study. Protocols reviewed and approved by the Institutional Review Board were adopted to ensure voluntary participation and confidentiality of survey responses. Both SF-36 and CES-D tests were administered together, and a 2-week deadline was imposed for survey completion. Participants were instructed so as to the purpose of coadministration of the surveys and asked to complete both questionnaires in one session. It was estimated through pilot-testing in a small group of older adults that the surveys took about 30 minutes to complete. In order to ensure uniformity in survey administration across the entire sample, participants were instructed to respond to the SF-36 survey first, followed by the CES-D questionnaire. Reminder e-mails were sent a week following the initial online dispatch of the survey instruments. The sample contained individuals with a range of background education and reported themselves to be in a good to excellent physical health.

### 2.2. Measures

The 36 items in SF-36 measure eight health concepts (or constructs) and health transition (physical function, role function, bodily pain, vitality, social functioning, role-emotional, mental health, general health, and health transition) [14]. The instrument also generates two summary measures of health, namely, physical component score (PCS) and mental component score (MCS), which together represent all the eight SF-36 domains. The scale items were scored using Likert's method of summated ratings, where multi-item scale score is computed by simply summing the scores assigned to item responses and by transforming scores to a 0–100 range. Higher scores indicate better health. In addition to eight domain scores, summary physical and mental component scores (PCS and MCS) were also computed. Cutoff scores of <52 on the mental health (MH) subscale were used to identify older adults at risk for depression. In order to facilitate easy data interpretation, the publishers of SF-36 survey tools recommend the use of normative data obtained from studies conducted in general populations, which are then used as “benchmarks” against which to compare observed scores and changes in scores [14]. This comparison is called norm-based interpretation of SF-36 data and enables ready interpretation of the burden of disease. For example, the lower the observed score for a patient from the norm is, the greater the burden of disease is. Useful published norms are available with adjustments for age, gender, and medical conditions [14].

The 20-item CES-D scale is designed to measure the depressive mood in the general population. The respondents are asked to rate the frequency, over the past week, of 20 symptoms of depression by choosing one of four response options ranging from “rarely or none of the time” to “most or all of the time.” Scores range from 0 to 60, where a score greater than 60 indicates “impairment.”

In addition to the two instruments, data on respondents' sociodemographics, medication use, and arthritis-related care were also gathered. The data analysis was carried out with the aid of SPSS (Version 11.0) statistical software package [25]. Descriptive univariate statistics were used to analyze crucial study variables and bivariate associations among different health status domains, and CES-D score was determined using Pearson's correlations, using a priori significance level of 0.05.

## 3. Results

 Survey responses were retrieved electronically following the 2-week deadline and stored in a spreadsheet format. The surveys were then appropriately coded according to the scoring guidelines recommended for SF-36 [14] and CES-D [13]. Incomplete surveys or questionnaires with missing values for more than half of the items on each subscale of the SF-36 questionnaire were discarded. Similarly, for the CES-D survey, those who failed to answer at least 17 of the 20 items were discarded. A response rate of 87% (*N* = 480) was achieved following elimination of incomplete and/or unusable surveys.  Table 1 describes sociodemographic characteristics of the sample. A majority of the older adults were white (95.5%), female (52.9%), married (70.9%), and belonged to the age group of 65–74 years (57.3%). About 42.3% of the sample (*n* = 203) reported having osteoarthritis, about 16.5% (*n* = 79) had rheumatoid arthritis, 12.7% osteoporosis (*n* = 61), and about 29% (*n* = 137) had diagnosis of other rheumatic conditions.  Table 2 provides information on health status scores of arthritic individuals on the eight SF-36 domains.  Table 3 describes group differences on summary component scores between male and female respondents and between two age groups. Male older adults performed better than females on both PCS and MCS dimensions. However, the difference was statistically significant (*P* < 0.05) between the two groups for only MCS scores. Respondents aged 75 and over exhibited poorer physical and mental component scores than the respondents in the younger age group. The potential range of the CES-D scale is 0–60; the range for our sample was 0–57.  Table 4 presents differences in CES-D scores on gender and age variables. According to the student *t*-test analysis, respondents aged 75 and over appeared to be significantly more depressed (*P* < 0.05) than the rest of the sample. No such differences were found between male and female elderly respondents. Using CESD cutoff score of >16, 16% (*n* = 76) of the respondents were found to be at risk for clinical depression, as opposed to only 4% (*n* = 19) of the subjects who scored below the cut point (<52) on the MH subscale of SF-36. As expected, a strong, negative correlation (*r* = −0.68, *P* < 0.05) was found between the CES-D total scores and MH subscale of SF-36. Further, analysis of variance (ANOVA) was performed to examine overall group differences on SF-36 variables and between different demographic subgroups to identify variables where differences originated (Table 5). Data showed significant group differences on PCS component scores among subgroups based on marital status, education, and personal income (*P* < 0.05). ANOVA showed that income and education contributed more to between-group differences than differences based on other sociodemographic factors. Significant differences on mental health status scores were obtained only for different levels of educational qualification (*P* < 0.05). We performed a series of partial correlations between depression and health status scores to investigate whether age was the factor in contributing to the relationship between health and depression. The correlations between depression and health were unaffected by age. Thus it appears that older age did not significantly contribute to this relationship.

## 4. Discussion

Based on the cutpoint defined for CES-D, we identified 16% of the older people as the group that is “very likely at risk for a disorder requiring further medical attention.” It must be noted here that these individuals probably had diagnosable psychiatric disorders and depression, even though diagnosis of depression *per se* might not be ruled out. The prevalence rates found in this study of 16% is similar to the rates found in the previous studies [26, 27]. The number we have witnessed here is significant and calls for primary care providers to be more vigilant when caring for elderly patients with arthritis, as depression seems to be largely undiagnosed in this group. According to one report, the prevalence of significant depressive symptoms in medically ill elderly and residents of long term-care residents may be as high as 50% [28]. The numbers we obtained in our study are not as alarming but do stress the need for early detection and diagnosis of depression and prompt treatment. Research demonstrates that depressed patients use more health care services than nondepressed patients [29]. Therefore, from health services perspective, primary care can be an appropriate setting for arthritis care so as to minimize unnecessary utilization of health care resources when arthritis is concomitant with depression in elderly individuals. One good primary care strategy might be to identify individuals having arthritis who exceed the threshold on the CES-D scale and provide them with appropriate care so as to minimize mental health services use and pharmacotherapy necessary to manage care.

Lower MH and MCS scores for both men and women aged 75 years and over showed that older adults in this group were more likely to report symptoms of depression than people in younger age groups. Further, older men and women with arthritis were more likely to report symptoms of depression than older men and women with no chronic condition, as evidenced by the comparison of our study population with the benchmarks obtained from the general elderly U.S. population. It appears that the relationship between physical health and functioning and depressive symptoms may have occurred as a result of association between items representing somatic symptoms on the CES-D scale and physical illness. It is not known if depression in our sample was caused by the physical illness alone, older age, or both. Even though depression can be a reaction to physical illness, it can also cause physical impairment due to what can be regarded as a cyclical relationship between the two. Given the nature of these complex interrelationships, it is often possible to overlook cases of depression in older people who might tend to attribute their depressive symptoms to physical illness alone. Available evidence seems to indicate that that physical illness and depression might be intricately linked. According to one study, for patients who were managed under a comprehensive depression management program comprising antidepressant medications and multiple sessions of psychotherapy, improved primary care treatment of depression for the elderly not only reduced depressive symptoms but also reduced arthritis pain and improved quality of life as well [30]. The special program developed in this study for patients with arthritis and concurrent depression produced significant improvement over the usual care group in functional impairment, general self-assessed health status, and quality of life. Improvement in both arthritis pain and depression among patients in the depression management program was seen at the same time, suggesting a strong relationship between the two outcomes. Because of the cross-sectional nature of our data, it is impossible to draw inferences concerning the direction of causality with regard to physical illness and depression in our study. From a clinical perspective, the best approach at treatment seems to be one that combines depression screening and a systematic, ongoing assessment of pain in patients with arthritis in order to maximize functional status and quality of life, especially when high comorbidity of arthritis and late-life depression exist.

The mental health subscale (MH) of the SF-36 and its cut-offs seem to perform somewhat satisfactorily in the detection of depression. However, the fact that MH scale identified fewer cases of depressive mood than a more reliable and valid CES-D raises questions about the effectiveness of SF-36 as an independent depression screener. One reason for the underestimation of depression in our sample by the SF-36 scale (compared to an established CES-D) may be that it contains fewer items that measure symptoms of depression. Therefore, underrepresentation of depression items in the instrument at this time makes it less preferable for the assessment of depression along with health status. It can be argued that SF-36 was not designed with a purpose to measure depression, even though the instrument, when originally conceived and developed, embodied many of the concepts represented by the well-known geriatric depression instruments available at the time, including CES-D [14]. We did not perform ROC analyses in this study to assess true positive (sensitivity) and false positive rates (specificity) of the full range of MH and its cutoffs in detecting depression. Doing so would have perhaps provided a more definite word on the utility of SF-36 in depression measurement. In absence of such efforts, however, caution should be exercised while using SF-36 as a sole screener for depression. At best, MH cutpoint scores should serve as first stage screen for further testing and not the final word on the diagnosis of depression. More research is warranted to explore possibilities for SF-36 to be used as a tool in the identification and treatment of depression. Results from this study can also help guide future research on predictors and risk factors of depression concurrent to a physical illness and the value of early detection and intervention in arthritis management.

We conclude that older individuals having arthritis are at a moderate risk of clinical depression based on their mental health status. From health services perspective, a significant decline in health in all the domains of arthritis-affected older persons, particularly in mental and social functioning domains, when compared to the general population should be an area of concern. Early recognition by primary care specialists of depressive symptomatology and its prompt treatment is essential to lessen the public health burden arising from the comorbidity with arthritis. More evidence is needed to support the use of SF-36 as a depression screener as it appears to perform inadequately compared to an established depression scale in the identification of population at risk for depression.

## Figures and Tables

**Table 1:** Demographic characteristics of elderly nationwide sample (*N* = 480).

Demographic variable	Response category	*n*	%
Age	65–74 yrs	275	57.3
	75+ yrs	205	42.7

Gender	Male	226	47.1
	Female	254	52.9

Race	White	444	95.5
	Black/African-American	12	2.6
	American native/Alaska native	3	0.6
	Asian/Pacific Islander	3	0.6
	Other	3	0.6

Region	Northeast	73	15.2
	Midwest	134	27.9
	South	148	30.8
	West	125	26.0

Marital status	Married	334	70.9
	Widowed	110	23.4
	Divorced	20	4.2
	Separated	2	0.4
	Never married	5	1.1

Education	Less than high school	56	11.7
	Some high school	29	6.1
	High school graduate/equivalent	109	22.8
	Some college	130	27.2
	Associate degree	14	2.9
	Bachelor's degree	89	18.6
	Master's degree	33	6.9
	Professional degree	10	2.1
	Doctorate degree	8	1.7

Pretax income	Below $35,000	160	34.3
	$35,000 or more	227	59.4

**Table 2:** Comparison of sample SF-36 domain scores with the norms for the general US population.

SF-36 Domains	Mean general US population score*	Mean US score (Ages 65–74 years )*	Mean US score (Ages 75 years and older)*	Mean arthritis sample score (65 years and older)
General health (GH)	71.95	62.56	56.66	63.25
Physical functioning (PF)	84.15	69.38	53.20	59.14
Role physical (RP)	80.96	64.54	43.28	53.09
Bodily pain (BP)	75.15	68.49	60.88	56.12
Vitality (VT)	60.86	59.94	50.41	53.20
Mental health (MH)	74.74	76.87	73.99	79.82
Social functioning (SF)	83.28	80.61	73.89	81.38
Role emotional (RE)	81.26	81.44	63.18	79.75

*Norms obtained from: Ware et al. [14].

**Table 3:** Demographic group differences on physical component summary (PCS) scores and mental component summary (MCS) scores.

SF-36 Domains	Demographic variable	Levels	*n*	Mean	*t*
PCS	Sex	Male	226	39.60	3.00*
		Female	254	36.64	
	Age	65–74	226	39.60	3.00*
		75+	254	35.51	

MCS	Sex	Male	226	49.69	0.74
		Female	254	48.76	
	Age	65–74	275	50.69	2.7*
		75+	205	47.19	

*P < 0.05; *N* = 480.

**Table 4:** Demographic group differences on center for epidemiological scale for depression (CES-D) scores.

Variables	Levels	*N*	Mean	*t*
Gender	Male	226	9.16	−0.849
	Female	254	9.76	

Age groups	65–74	275	8.71	−2.564*
	75+	205	10.51	

*P < 0.05; *N* = 480.

**Table 5:** Analysis of variance (ANOVA) examining group differences on SF-36 domain scores and summary component scores.

Demographic variable	SF-36 domain	*F* value	Significance
Race^a^	PCS	1.224	0.3
	MCS	1.093	0.359
	General health	1.091	0.36
	Mental health	1.689	0.151

Region^b^	PCS	0.18	0.91
	MCS	0.457	0.713
	General health (GH)	0.402	0.752
	Mental health (MH)	0.418	0.74

Marital status^c^	PCS	5.759	0.000*
	MCS	0.883	0.474
	General health (GH)	1.771	0.133
	Mental health (MH)	1.059	0.376

Education^d^	PCS	2.893	0.004*
	MCS	2.608	0.008*
	General health (GH)	2.083	0.036*
	Mental health (MH)	2.379	0.016*

Income^e^	PCS	2.037	0.01*
	MCS	1.562	0.076
	General health (GH)	1.891	0.02*
	Mental health (MH)	1.248	0.228

*P < 0.05; *N* = 480.

PCS: Physical Component Summary score; MCS: Mental Component Summary score.

^a^White, African-American, Native American/Alaskan, Asian/Pacific Islander, Other.

^b^Northeast, Midwest, South, West.

^c^Married, widowed, divorced, separated, never married.

^d^Less than high school, some high school, high school graduate or equivalent, some college, associate degree, bachelor's degree, master's degree, professional degree, doctorate degree.

^e^ <$5000, $5000–$7499, $7500–$9999, $10000–$12499, $12500–$14999, $15000–$19999, $20000–$24999, $25000–$29999, $30000–$34999, $35000–$39999, $40000–$49999, $50000–$59999, $60000–$74999, $75000–$84999, $85000–$99999, $100000–$124999, >$125000.
